# Supplementary material for: Causal Relationship between Aging and Anorexia Nervosa: A White-Matter-Microstructure-Mediated Mendelian Randomization Analysis
Source: Biomedicines. 2024 Aug 16;12(8):1874. doi: 10.3390/biomedicines12081874 (PMC11351342; doi:10.3390/biomedicines12081874)
Supplement: Supplementary file 1 [file biomedicines-12-01874-s001.zip › Supplementary Figure/Figure S4/Singe SNP plot of MR analysis for TL to ubm-b-1667.pdf]

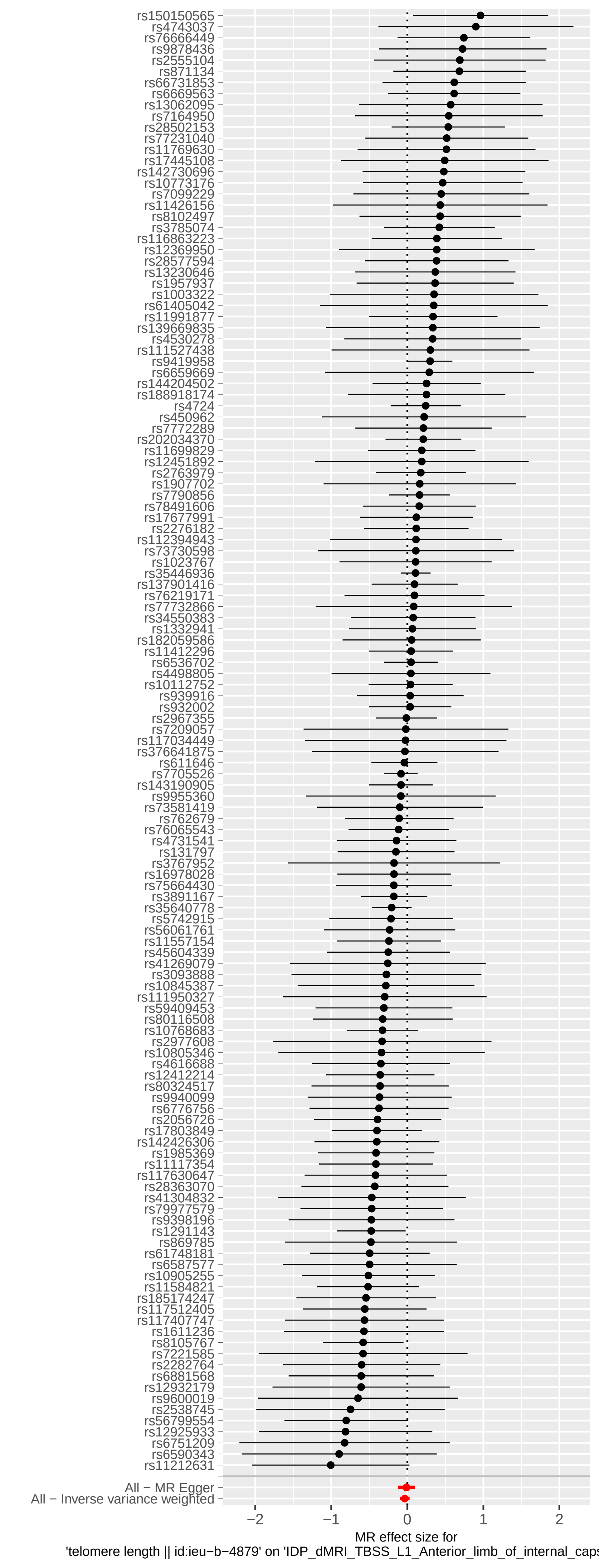
rs150150565
rs4743037
rs76666449
rs9878436
rs2555104
rs871134
rs66731853
rs6669563
rs13062095
rs7164950
rs28502153
rs77231040
rs11769630
rs17445108
rs142730696
rs10773176
rs7099229
rs11426156
rs8102497
rs3785074
rs116863223
rs12369950
rs28577594
rs13230646
rs1957937
rs1003322
rs61405042
rs11991877
rs139669835
rs4530278
rs111527438
rs9419958
rs6659669
rs144204502
rs188918174
rs4724
rs450962
rs7772289
rs202034370
rs11699829
rs12451892
rs2763979
rs1907702
rs7790856
rs78491606
rs17677991
rs2276182
rs112394943
rs73730598
rs1023767
rs35446936
rs137901416
rs76219171
rs77732866
rs34550383
rs1332941
rs182059586
rs11412296
rs6536702
rs4498805
rs10112752
rs939916
rs932002
rs2967355
rs7209057
rs117034449
rs376641875
rs611646
rs7705526
rs143190905
rs9955360
rs73581419
rs762679
rs76065543
rs4731541
rs131797
rs3767952
rs16978028
rs75664430
rs3891167
rs35640778
rs5742915
rs56061761
rs11557154
rs45604339
rs41269079
rs3093888
rs10845387
rs111950327
rs59409453
rs80116508
rs10768683
rs2977608
rs10805346
rs4616688
rs12412214
rs80324517
rs9940099
rs6776756
rs2056726
rs17803849
rs142426306
rs1985369
rs11117354
rs117630647
rs28363070
rs41304832
rs79977579
rs9398196
rs1291143
rs869785
rs61748181
rs6587577
rs10905255
rs11584821
rs185174247
rs117512405
rs117407747
rs1611236
rs8105767
rs7221585
rs2282764
rs6881568
rs12932179
rs9600019
rs2538745
rs56799554
rs12925933
rs6751209
rs6590343
rs11212631
All − MR Egger
All − Inverse variance weighted
−2
−1
0
1
2
MR effect size for
'telomere length || id:ieu−b−4879' on 'IDP_dMRI_TBSS_L1_Anterior_limb_of_internal_caps
